# Randomized Controlled Trial comparing the effectiveness of structured-play (ENGAGE) and behavior management (TRIPLE P) in reducing problem behaviors in preschoolers

**DOI:** 10.1038/s41598-019-40234-0

**Published:** 2019-03-05

**Authors:** Dione Healey, Matthew Healey

**Affiliations:** 1 Department of Psychology, Box 56, University of Otago, Dunedin, 9054 New Zealand; 2 Information Technology Services, Box 56, University of Otago, Dunedin, 9054 New Zealand

## Abstract

Children’s behavior problems are a growing concern in our society; and are associated with a wide array of adverse lifespan outcomes. Current treatments are not without limitations and while effective for many, do not help all children. As such, additional treatment options are required. Sixty families of children aged 3 and 4 years participated. In order to participate, children needed to have a T-score of 60 or above on the parent rated Hyperactivity subscale on the BASC-2. Families were randomly assigned to either a structured play-based intervention (ENGAGE; n = 29), or to the current gold standard treatment for preschool behavior problems, behavior management (Triple P; n = 31). This trial has been registered with the Australian New Zealand Clinical Trials Registry (ANZCTR); trial number ACTRN12617001432303; trial web address: http://www.anzctr.org.au/ACTRN12617001432303.aspx; date registered; 10/10/2017. ENGAGE was found to be as effective as Triple P in reducing parent-rated problem behaviors in pre-schoolers (i.e., Hyperactivity, Attention Problems, and Aggression); with gains maintained over a 12-month follow-up period, for both interventions. These findings indicate that structured play is an equally effective alternative way to manage difficult behavior in preschoolers and compliments our current treatment options.

## Introduction

All young primates play. Play fosters cognitive, physical, social, and emotional wellbeing and is essential for optimal child development^1^. Play is so important that the United Nations High Commission for Human Rights deems it to be the right of every child^2^. Despite the obvious benefits of play, children are spending less and less time at play^3^. Working parents, hurried lifestyles, instant entertainment on devices like smart phones, and increased emphasis on academics all reduce play time^2^. In step with this reduction in play, mental health problems are constantly increasing worldwide^3–6^. Could it be that simply slowing down and spending more time playing with our children is the answer to reducing these increasing rates of mental health problems?

Children learn self-regulation through play^7^. For example, in structured games they need to wait their turn, plan their next move, focus on the ball, and manage frustration when things don’t go their way. The inability to self-regulate has long been associated with behavioral, emotional, social, and learning difficulties in childhood; followed by later criminality, poverty, poor job performance, and physical and mental health difficulties^8–11^. Poor self-regulation in childhood is evidenced by hyperactivity, inattention, and aggression^12^. These attributes are associated with childhood disorders such as Attention Deficit Hyperactivity Disorder and Conduct Disorder; but even without meeting criteria for these disorders lower self-regulatory skills are associated with higher social, emotional, behavioral, and academic difficulties in children^9,13,14^.

The current gold standard treatments for these childhood behavioral difficulties are behavior management and in more extreme cases, medication. While both are effective, they are not without their limitations^15^. For example, medication compliance is often low^16^, medications can have negative side effects, and not all children respond well to them^17^. Behavioural interventions are often more palatable for parents than medication, but are generally more difficult to implement, can be quite costly, and are typically less effective than medications in the more severe cases^18^.

Given the high rates of behaviour problems in children, the fact that these are increasing, and that not all children are benefiting from our current treatments, we need to continue to diversify our offerings in order to increase the chances of successfully treating these problems early on, and hopefully changing the life course trajectory for these at-risk children.

Structured play appears to be a viable additional treatment option. While the research is still in its infancy, the studies available show that structured play is a promising approach to improving self-regulation in young children. Three key studies have looked at its effect on levels of hyperactivity, inattention, and aggression and all have shown that it leads to significant reductions in these behaviours^12,19,20^. However, this approach has not yet been directly compared to the current gold standard (i.e, medication or behaviour management). Thus, the aim of this study was to compare a structured play-based intervention, ENGAGE (Enhancing Neurobehavioural Gains with the Aid of Games and Exercise)^19^ to a strongly evidence based, highly effective behaviour management programme, Triple P (Positive Parenting Programme)^21^. ENGAGE involves parents playing a range of common games with their children in a structured way for half an hour a day (e.g., puzzles, ball games, musical statues, blocks, skip rope. See Table 1 for a description of all games). The games all require aspects of self-regulation (e.g., waiting your turn, inhibiting a response, regulating emotion). Triple P functions to improve self-regulation by, for example, providing clear and logical consequences to guide behaviour and using techniques such as quiet time and time out to allow children space and time to self-soothe.

**Table 1:** List of the games included in the ENGAGE programme.

*Behavioural Self-Regulation* Musical Statues	Move around while music is playing. Freeze when the music stops
Animal Speeds	Regulate the speed of your activity between small, moderate, and fast speeds
Skipping	Regulate the speed at which skipping occurs
Ball and Spoon Race	Hold a spoon with a ball on it and move from one place to another at varying speeds
Simon Says	Repeat an action if Simon says to do “this” but do not repeat an action when Simon says do “that”
Snap	Card game where if identical cards are placed down in a sequence, you place your hand on the cards and say “Snap”
Hop Scotch	Aim a token for the correct number in the sequence and hop to that number
Drawing	Improve fine motor control through drawing
Leap Frog	Remain still while other jump over you and wait until it is your turn to jump over others
*Cognitive Self-Regulation* Copy Me	Watch a sequence. Then repeat the sequence from memory
Object Copy	Observe and structure being built. Re-create the structure from memory
Ball Games	Various games that involve having to focus on the ball and catch it
Puzzles	Complete puzzles
Cups Memory	Remember which cups have been picked up and the token underneath removed
Card Memory	Remember where the matching card is and turn over 2 matching cards to collect a pair
Beading	Thread beads either from memory of a sequence or according to various changing rules
Tracking Memory	Watch cups being moved around on a table and afterward identify which one has the token under it
List Memory	Remember a list that is continually being added to
Sorting	Sort various materials according to different rules
*Emotional Self-Regulation* Relaxation	Various exercises involving tension and relaxation of muscles
Deep Breathing	Learn to breathe in by filling your stomach with air (like a balloon) and then breath out slowly
Yoga	Basic yoga exercises to encourage controlled relaxation

We hypothesised that ENGAGE would be equally as effective as Triple P given that the effect-sizes for both behavioural management and play-based interventions in past studies have been similar^12,22–25^.

## Method

### Participants

Sixty families, living in Dunedin, New Zealand, with children aged 3–4 years, participated. To meet criteria for participation, parents had to have rated their child’s hyperactivity at the 84^th^ percentile or above (i.e., T-score of 60 or above) on the Behavior Assessment System for Children (BASC-2)^26^ and be able to attend weekly intervention sessions at our research centre. Most of the participating children were of European descent (83%), with a further 11% of mixed European/New Zealand Māori descent, one of Maori, and one of Japanese descent. Parents of participants spanned the full range of educational levels from “some high school but did not complete” (n = 9) to “University degree” (n = 26), with the remainder (n = 25) falling between these two extremes. They also spanned the full range of income levels which ranged from 1 = less than $20,000 to 10 = more than $100,000). Once allocated to their intervention groups, demographic information was compared across groups with no significant differences apart from father’s education level (see Table 2).

**Table 2:** Descriptive statistics pertaining to key demographic variables.

Demographic Variable	ENGAGE M(SD) Range	Triple P M(SD)	*t*	*p*
Gender	22 boys	23 boys	−0.147	0.884
Age in months	45.55 (6.74) 36–57	45.42 (6.52) 36–59	0.077	0.939
Mother’s highest level of education	3.52 (1.46) 1–5	3.81 (1.47) 1–5	−0.765	0.447
Father’s highest level of education	3.93 (1.60) 1–7	3.00 (1.77) 1–5	2.131	0.037
Household income	5.73 (2.59) 1–10	6.00 (2.65) 1–10	−0.377	0.707

Participants were recruited through advertisements in local newspapers and a database of participants held in the Department of Psychology at the University of Otago, who were recruited at birth and parents had agreed to be contacted for research studies. Parents were asked to contact us if they believed their child to be difficult to manage (i.e., very active and impulsive and have difficulties with self-regulation). There were no gender or ethnic restrictions to participation, but both children and parents were required to be English-speaking, and children needed to attend a preschool or day care programme. Exclusionary criteria also included an estimated Full-Scale IQ score of less than 80, as measured by a trained postgraduate psychology student using the verbal and non-verbal routine subtests of the Stanford Binet^27^; a pervasive developmental disorder; a diagnosed neurological disorder; or those who were taking systemic medication for a chronic medical condition.

### Experimental Design

The CONSORT diagram (see supplementary files) details the recruitment and allocation flow of participants in this study. A total of 125 individuals responded to our recruitment advertisements. Of these, sixty respondents did not meet inclusion criteria; for 30 parents did not rate their child’s Hyperactivity at a T-score of 60 or above on the BASC-2. An additional 30 were unable to participate due to reasons such as: unable to attend the allocated group intervention time, living in another city numerous hours’ drive away and unable to travel for the weekly group sessions, unable to be contacted again after initial expression of interest in the study, did not attend scheduled initial assessment sessions and were unable to reschedule. Sixty-five respondents were eligible for study participation. Five of these attended an initial assessment session but did not complete the interventions as they either had a change in work hours which meant they were no longer able to attend the weekly session, or they reported that they were too now busy to commit to weekly attendance. This left a total of 60 participants who attended the initial assessment sessions and the interventions.

Participants were initially randomly assigned to either a waitlist (n = 32) or non-waitlist (n = 33) group; however, 5 of these (2 waitlisted and 3 non-waitlisted) did not begin the interventions for reasons described above. Randomisation occurred using computer generated randomization conducted by our research administrator who managed recruitment for this study. Our sample size was based on the plan that thirty participants would receive ENGAGE (90% power to predict pre-post intervention differences, based on ENGAGE open trial data^12^ and 30 would receive Triple P (90% power to detect pre-post intervention differences, based on published Triple P results; 22).

The waitlist group were assessed at baseline and then again 8 weeks later. They did not receive any intervention over this time and the data collected was used as a control for treatment effects. Following the waitlist period, the waitlisted participants were randomly assigned to either ENGAGE (n = 15) or Triple P (n = 15). The non-waitlist group were directly randomly assigned to either ENGAGE (n = 14) or Triple P (n = 16). Thus, a total of 29 families received ENGAGE (15 of them had also undergone waitlist assessments); and 31 families received Triple P (15 of them had also undergone waitlist assessments). There were no significant group differences in age, ethnicity, parent highest education level or any of the key study measures (i.e., parent-ratings, teacher-ratings and neurocognitive test scores) for those assigned to ENGAGE versus Triple P.

### Procedure

Upon responding to recruitment advertisements, parents were initially informed about the goals of the study over the phone. They were then sent information sheets and consent forms, along with BASC-2 questionnaires for parents and teachers to complete. Parents were asked to pass the BASC-2 on to their child’s teacher along with a self-addressed envelope in which to send the completed form back to the researches. Both parents and teacher provided informed consent in writing. Once the completed forms had been returned, those who met the entry criteria were invited to attend a baseline assessment session at our university research centre. During this session, the children were administered subtests of NEPSY-2^28^, as well as the Head-Toes-Knees-Shoulders task (10; all described below) by a trained postgraduate psychology student, to assess functioning within neurocognitive domains associated with self-regulation. Those in the waitlist group were reassessed on the same parent, teacher, and child neurocognitive measures 8 weeks later.

The intervention began the week following either the initial baseline session (non-waitlist) or the second waitlist assessment (8 weeks later). Both interventions were conducted by the same two clinical psychologists with specialized training in ENGAGE and formal accreditation as Triple P group intervention providers. Both interventions are manualised and protocols were strictly adhered to. The clinical psychologists were not aware of the specific research hypotheses regarding the two interventions and were not involved in the study in any role other than group facilitator. One clinical psychologist ran the majority of the parent groups (10 groups: 5 ENGAGE groups with a total of 25 participants; and 5 Triple P groups with a total of 21 participants). Trained postgraduate students ran the child groups concurrently with the parent groups.

#### Enhancing Neurobehavioural Gains with the Aid of Games and Exercise (ENGAGE)

This intervention involved parents and children attending a weekly 1.5 hour group session for five weeks, followed by two weeks of individual phone calls and then a final group session in the 8^th^ week. While attending the intervention sessions, a group of up to six parents were together in one room where they were taught a new set of games each week and asked to play them with their children for 30 minutes a day. All of the games targeted neurocognitive areas known to be associated with self-regulation (see Table 1 for a list of all the games along with a brief description). In an adjacent room, their children were taught the same games by a trained postgraduate psychology student so as to familiarize them with the games, engage them in the activities, and make it easier for parents to introduce the games to them at home. When all of the games had been taught, parents were encouraged to continue to play the games, increasing the complexity of the games as their child developed the skills. They then received individual phone calls once a week for two weeks (Session 6 & 7) where they were aided in further individualizing the program to their own child and any issues or questions were addressed by the clinical psychologist who had been facilitating the groups. Following this a final group session was held (session 8). This was in the form of a booster session and focused on maintenance of the program over time.

For ENGAGE there were a total of six groups run; three of them had four families in them; two of them had six families in them; and one had five families in it.

The Standard Group Triple P programme was used in this study. This is also an 8-week program. For the first 4 weeks (Sessions 1–4) a group of up to six parents attended a weekly 1.5 hour session where they were taught 17 core child management strategies. These were divided into 10 strategies used to promote positive development (e.g., talking with children, physical affection, spending quality time together, setting a good example) and 7 strategies for managing misbehaviour (e.g., setting rules, ignoring unwanted behaviours, time-out). After 4 weeks, parents received 3 weekly phone calls (Sessions 5–7) designed to help parents continue to implement the strategies taught in sessions 1–4. In the eighth week (Session 8) of the program parents attended a final group session focused on maintenance of the program.

For Triple P there were seven groups run in total; three of them had four families in them, two had five families in them, one had three families, and one had six families.

### Ethical Approval

This study received ethical approval from the University of Otago Human Ethics Committee prior to commencement. Informed consent was obtained from parents and teachers taking part; and assent was obtained from participating young children. While conducting this study, we have complied with all ethical standards of the American Psychology Association.

### Measures

#### Behavioral Measures

Behavior Assessment System for Children (BASC-2*; 26*) is a well-validated and normed scale designed to assess wide ranging areas of child functioning, as rated by parents and teachers. Of particular interest to this study were the Hyperactivity, Aggression, and Attention Problems subscales of this measure as they are indicative of self-regulatory ability. Parents and teachers were asked to complete the BASC-2 either four times (baseline, post-intervention, 6, and 12 months post-intervention); or 5 times if they were in the waitlist condition (waitlist, baseline, post-intervention, 6, and 12 months post-intervention)

### Neurocognitive Measures

Stanford Binet^27^ is a widely-used test of intelligence with well-established psychometric properties. For this study, a valid short form of the test, which included the two routing subtests, was used to estimate IQ, as participants with an IQ score below 80 were not eligible to participate.

Developmental Neuropsychological Assessment (NEPSY; 28); is a test battery designed to assess numerous areas of neuropsychological functioning in children. It is well-normed, reliable, and appropriate for use with 3–4 year old children. Three tests from this battery were administered at waitlist, baseline, post-intervention, 6 and 12 months follow-up to assess targeted areas of neuropsychological functioning associated with attention, memory, and inhibitory control (cognitive functions associated with self-regulation). These included the Statue subtest, which measures inhibitory control; Comprehension of Instructions, which assesses language and working memory; and Visuomotor Precision, which assesses motor and inhibitory control.

Head-Toes-Knees-Shoulders^10^ is a measure of inhibitory control designed for use with young children, and was used as a measure of behavioural self-regulation. The task requires children to provide an opposite response to what is said (e.g., if asked to touch their head they should touch their toes). It was also administered at waitlist, baseline, post-intervention, 6 and 12 months follow-up.

### Data Analysis

For both behavioural and neurocognitive measures data were analysed by applying analysis of variance (Anova) on conditional growth models using the statistical software R and the libraries lme4, car, multcomp and lme^29^. Given the known age effects for the measures used (i.e., hyperactivity, attention problems, and aggression all tend to reduce with age), all models controlled for the age of participant at the time of interaction. Effect sizes (hedge’s g) were also calculated for multiple comparisons between key time points.

## Results

### Treatment compliance

To assess the degree to which parents used the intervention strategies at home, they were asked to complete weekly diaries. For ENGAGE they recorded how much time they spent playing the games each day. Parents had been encouraged to spend half an hour a day playing the games and on average parents reported spending 29.81 minutes a day playing the games (SD 7.75) with average time ranging from (18–45 mins). They also reported playing the games on an average of 5 days per week (SD = 1.10) with the range of days per week ranging from 2–7.

For Triple P parents were asked to use the strategies taught whenever there was an opportunity to do so in response to their child’s behaviour. On average parents reported using the strategies 10.57 (SD = 4.26) times a week (with a range from 3–18). Of the times when they could have used the strategies, on average parents reported using them 76.8% of the time (SD 13.35); with a range from 46–98%).

These results show that parents were highly engaged in the interventions and were frequently using the strategies as instructed during the interventions.

### Parent Ratings

**Figure 1:**
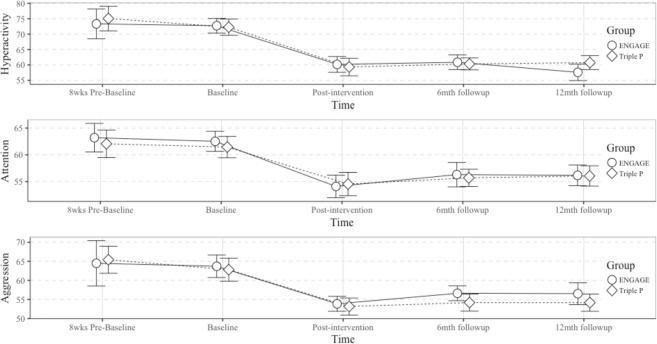
Changes in Hyperactivity, Attention Problems, and Aggression (BASC-2; T-scores) within and between groups over time; controlling for age.

To examine whether there was a reduction in hyperactivity, attention problems, and aggression ratings by parents on the BASC-2 for both treatments, across the five time periods, ANOVAs, controlling for age, were conducted on the mixed effects models for each measure and on a combined group set. No statistically significant differences between the two groups, ENGAGE and Triple P were observed (see Fig. 1 and Table 3).

**Table 3:** Parent Ratings of children’s Hyperactivity, Aggression and Attention Problems as BASC-2 T-scores, within and between groups, over 5 time points, controlling for age.

	Waitlist (WL)	Baseline (BL)	Post intervention (PI)	6 month follow-up	12 month follow-up	Main effect Group	Simple effect Time	Multi. comp. WL v BL	Multi. comp BL v PI	Multi. comp. PI v 12mo FU
	M (SD)	M (SD)	M (SD)	M (SD)	M (SD)	Chi-sq.	Chi-sq.	Z. (Hedge’s g)	Z. (Hedge’s g)	Z. (Hedge’s g)
Hyperactivity										
Engage	73.33 (8.77)	72.72 (6.21)	60.17 (6.71)	60.86 (6.24)	57.59 (7.03)	0.03 N.S	76.76***	0.74 (0.58)	6.92*** (1.91)	1.02 (0.37)
Triple P	75.07 (7.23)	72.26 (7.27)	59.32 (7.78)	60.32 (5.30)	60.74 (6.13)		58.98***	1.71 (0.3 8)	7.34*** (1.70)	−0.59 (−0.20)
Attention										
Engage	63.20 (4.81)	62.52 (4.93)	54.10 (5.57)	56.31 (6.00)	56.17 (5.09)	0.05 N.S	76.75***	1.14 (0.14)	6.04*** (1.58)	−1.24 (−0.38)
Triple P	62.07 (4.64)	61.45 (5.46)	54.55 (5.95)	55.71 (4.40)	56.06 (5.16)		28.93***	0.23 (0.12)	5.05*** (1.19)	−0.44 (−0.26)
Aggression										
Engage	64.47 (10.71)	63.69 (7.75)	53.86 (5.15)	56.62 (5.11)	56.52 (5.11)	0.83 N.S	45.29***	1.53 (0.08)	6.14*** (1.47)	−1.47 (−0.51)
Triple P	65.40 (6.40)	62.81 (8.24)	53.16 (6.09)	54.19 (6.13)	54.16 (6.13)		36.03***	1.00 (0.34)	5.33*** (1.32)	−0.16 (−0.16)

*p < 0.05; **p < 0.01; ***p < 0.001.

ENGAGE: WL n = 15; BL n = 29; PI n = 29; 12mo FU n = 29.

Triple P: WL n = 15; BL n = 31; PI n = 31; 12mo FU n = 31.

As shown in Table 3, after controlling for age, we found significant effects of time within group on all three behavioral measures, for ENGAGE (Hyperactivity: *Chi-sq* = 76.76, *p* < 0.001, Attention: *Chi-sq* = 76.75, *p* < 0.001, and Aggression: *Chi-sq* = 45.29, *p* < 0.001), and Triple P (Hyperactivity: *Chi-sq* = 58.98, *p* < 0.001, Attention: *Chi-sq* = 28.93, *p* = 0.00, Aggression: *Chi-sq* = 36.03, *p* < 0.001).

Adjusted (Tukey) multiple comparisons between waitlist and baseline showed no statistically significant difference between the time points for all measures in each group; indicating that children do not simply improve on these measures over time without treatment. Similarly effect sizes for the waitlist to baseline comparisons were mostly trivial (i.e., below 0.2) or small; with one medium effect for the ENGAGE group hyperactivity scores.

For comparisons between baseline and post-intervention, adjusted (Tukey) multiple comparisons revealed statistically significant differences between the time points for all measures in both groups; indicating improvements for both treatment groups. This is corroborated by effect sizes which were large for all measures.

For comparisons of post-intervention and 12-month follow-up no statistically significant differences were found; indicating that treatment gains were maintained over the 12-month follow-up period. Again, this was borne out in the effect sizes which were all negligible or small, apart from a medium effect for Aggression in the ENGAGE group which had increased at 12 month follow-up.

### Teacher Ratings

To examine whether there was a reduction in hyperactivity, attention problems, and aggression ratings by teachers on the BASC-2, for both treatments, across the five time periods, ANOVAs, controlling for age, were conducted on the mixed effects models for each measure and on a combined group set. No statistically significant differences between the two groups, ENGAGE and Triple P were observed (see Table 4).

**Table 4:** Teacher Ratings of children’s Hyperactivity, Aggression and Attention Problems as BASC-2 T-scores, within and between groups, over 5 time points, controlling for age.

	Waitlist (WL)	Baseline (BL)	Post intervention (PI)	6 month follow-up	12 month follow-up	Main effect Group	Simple effect Time	Multi. comp. WL v BL	Multi. comp. BL v PI	Multi. comp. PI v 12moFU
	M (SD)	M (SD)	M (SD)	M (SD)	M (SD)	Chi-sq.	Chi-sq.	Z. (Hedge’s g)	Z. (Hedge’s g)	Z. (Hedge’s g)
Hyperactivity										
Engage	54.00 (7.52)	54.64 (4.67)	52.32 (4.13)	53.08 (6.81)	54.16 (8.48)	0.43 N.S	25.33***	−3.14* (−0.10)	1.86 (0.52)	−1.62 (−0.26)
Triple P	52.80 (5.71)	52.75 (5.40)	48.71 (3.04)	50.69 (5.05)	52.81 (5.14)		30.97***	2.88* (0.01)	2.99*** (0.90)	−0.88 (−0.90)
Attention										
Engage	51.64 (9.10)	52.56 (5.48)	50.32 (4.72)	50.67 (4.70)	51.84 (8.80)	0.88 N.S	6.56 N.S	−0.71 (−0.13)	2.35 (0.43)	−0.70 (−0.21)
Triple P	49.60 (6.53)	49.68 (5.46)	46.17 (4.22)	47.79 (5.19)	51.19 (6.67)		41.98***	2.96* (−0.13)	2.03 (0.70)	−3.62** (−0.88)
Aggression										
Engage	55.07 (7.55)	54.20 (5.15)	53.45 (5.75)	53.38 (6.38)	52.68 (7.31)	0.00 N.S	1.64 N.S	−1.19 (0.14)	0.21 (0.14)	−0.19 (0.14)
Triple P	57.33 (7.54)	57.14 (8.54)	50.92 (5.65)	52.69 (6.82)	54.04 (5.71)		36.49***	4.09*** (0.02)	2.79* (0.85)	−0.89 (−0.54)

As shown in Table 4, significant effects of time were observed for Hyperactivity for both ENGAGE (*Chi-sq* = 25.33, *p* < 0.001) and Triple P (*Chi-sq* = 30.97, *p* < 0.001); and for Attention Problems (*Chi-sq* = 41.98, *p* < 0.001 and Aggression *Chi-sq* = 36.49, *p* < 0.001, for Triple P only. However, these effects are most prevalent within the waitlist to baseline comparisons where significant reductions in Hyperactivity were found for the ENGAGE group and significant reductions in Hyperactivity, Attention Problems, and Aggression were found for the Triple P group. This suggests that without any intervention, the teachers in the ENGAGE group reported reductions in hyperactivity and the teachers in the Triple P group reported improvements across all three behavioural measures. The teachers in the Triple P group reported further reductions in Hyperactivity and Aggression post-intervention and for Attention at 12-month follow-up. However, given the significant improvements reported by these teachers in the waitlist to baseline phase it is impossible to know whether the later reductions in teacher behavioural ratings are related to treatment effects.

Also, important to note is that mean ratings by teachers in both treatment groups, and across all three behavioural measures (Hyperactivity, Attention problems, and Aggression) at waitlist and baseline were all within the normal range and as such the small improvements seen are all within the normal range and not of clinical significance.

Effect sizes for the various time point comparisons were variable with a consistent pattern of the Triple P group showing larger improvements from baseline to post-intervention; but also larger increases in behaviour from post-intervention to 12 month follow-up; suggesting less maintenance of treatment gains over time. However, as mentioned above the fact that most of the scores were within the normal range it is difficult to draw strong conclusions with regard to true treatment effects.

### Neurocognitive functioning

To examine whether there were any improvements in neurocognitive functioning, for both treatments, across the five time periods, ANOVAs, controlling for age, were conducted on the mixed effects models for each measure and on a combined group set. Again, no statistically significant differences between the two groups, ENGAGE and Triple P were observed (see Table 5).

**Table 5:** Within and between group comparisons in children’s neuropsychological test scores over five time points, controlling for age.

	Waitlist (WL)	Baseline (BL)	Post intervention (PI)	6month follow-up	12month follow-up	Main effect Group	Simple effect Time	Multi. comp. WL v BL	Multi. comp BL v PI	Multi. comp. PI v 12moFU
	M (SD)	M (SD)	M (SD)	M (SD)	M (SD)	Chi-sq.	Chi-sq.	Z. (Hedge’s g)	Z. (Hedge’s g)	Z. (Hedge’s g)
Comprehension of instructions										
Engage	14.53 (2.87)	13.45 (2.29)	14.80 (2.12)	15.93 (2.18)	18.71 (2.31)	2.25 N.S	20.96***	−0.10 (0.42)	−2.02 (−0.06)	−3.58*** (−1.74)
Triple P	14.60 (2.96)	14.42 (2.15)	16.42 (1.97)	17.43 (1.88)	19.75 (2.26)		122.69***	−3.03* (0.07)	−3.46** (−1.00)	−6.35*** (−1.55)
Visuo-motor precision errors										
Engage	127.80 (26.10)	146.00 (49.29)	120.41 (47.02)	103.52 (38.31)	96.39 (51.30)	3.85 N.S	10.65*	−0.20 (−0.42)	1.96 (0.52)	−1.17 (0.48)
Triple P	129.60 (55.14)	129.35 (54.46)	93.84 (48.62)	93.33 (36.40	69.87 (46.82)		16.61**	0.22 (0.01)	2.30 (0.68)	−1.84 (−0.06)
Statue										
Engage	14.31 (5.16)	15.75 (5.67)	19.10 (7.19)	15.66 (5.73)	17.75 (6.21)	0.10 N.S	4.85 N.S	0.08 (−0.26)	−0.93 (−0.51)	−1.87 (−0.20)
Triple P	14.73 (7.77)	14.35 (7.06)	17.35 (7.14)	16.80 (6.09)	21.13 (6.20)		6.83 N.S	−1.17 (0.05)	−1.01 (−0.42)	−1.64 (−0.56)
HTKS										
Engage	7.71 (8.17)	7.96 (6.87)	8.89 (7.19)	14.48 (9.86)	24.75 (10.65)	1.10 N.S	22.50***	−2.27 (−0.03)	0.27 (−0.13)	4.38*** (−1.72)
Triple P	6.80 (11.15)	11.71 (9.87)	11.97 (9.06)	18.66 (10.22)	26.48 (14.45)		13.11**	−2.45 (−0.47)	1.36 (−0.03)	−2.74*** (−1.19)

*p < 0.05; **p < 0.01; ***p < 0.001.

ENGAGE: WL n = 15; BL n = 29; PI n = 29; 12mo FU n = 2.

Triple P: WL n = 15; BL n = 31; PI n = 31; 12mo FU n = 31.

As shown in Table 5, after controlling for age, we found significant effects of time within group on three of the four cognitive measures, for ENGAGE (Comprehension of Instructions: *Chi-sq* = 20.96, *p* < 0.001, Visuomotor Precision Errors: *Chi-sq* = 10.65, *p* < 0.05, and Heads-Toes-Knees-Shoulders (HTKS): *Chi-sq* = 22.50, *p* < 0.001), and Triple P (Comprehension of Instructions: *Chi-sq* = 122.69, *p* < 0.001, Visuomotor Precision Errors: *Chi-sq* = 16.61, *p* < 0.01, and Heads-Toes-Knees-Shoulders (HTKS): *Chi-sq* = 13.11, *p* < 0.01). No significant effects were found for either group on the Statue task.

Adjusted (Tukey) multiple comparisons between waitlist and baseline showed a statistically significant difference between the time points for Comprehension of Insturctions for the Triple P group; indicating that these children improved on this measures over time without treatment.

For comparisons between baseline and post-intervention, adjusted (Tukey) multiple comparisons again revealed statistically significant differences between the time points for Comprehension of Instructions, for the Triple P group; however given the improvement seen following the waitlist period, it is impossible to know whether the later improvements are related to treatment effects; especially given that children are doing the exact same task at each time point and therefore there is a high likelihood of practice effects.

For comparisons of post-intervention and 12-month follow-up; adjusted (Tukey) multiple comparisons again revealed statistically significant differences between the time points for Comprehension of Instructions for Triple P and this time for ENGAGE as well. Similarly, both groups showed improved scores on the HTKS task. As above, it is difficult to know whether these are treatment or practice effects.

Effect sizes for the various time point comparisons were variable with no consistent pattern within or between the intervention groups.

## Discussion

The aim of this study was to compare the effectiveness of a novel play-based intervention designed to improve self-regulatory skills in at-risk pre-schoolers, to that of behavioural management, a well validated, highly effective, long standing, treatment approach which is the current gold-standard psychological intervention for behavioural problems in young children.

Despite its vastly different approach, overall ENGAGE was found to be *as effective* in improving the children’s behaviour as Triple P, with reductions in hyperactivity, inattention, and aggression to within the typical range for their age at post-intervention; and maintained for 12 months afterward; according to parent report. These results replicate those of past studies showing that both ENGAGE^8^ and Triple P^16^ are effective treatments for reducing behavioural problems in young children. A significant strength of the current study is that we were able to maintain a 100% retention rate for parent ratings and child neurocognitive test scores (apart from the final 12 month follow-up neurocognitive testing session where one family in the ENGAGE group had moved away and was unable to attended the session, but did return the parent ratings by mail). Longitudinal studies are often hampered by missing data but this was not the case in the current study.

Parent report is limited by potential bias as the parents were active participants in the intervention. To overcome this, we collected two objective sources of information with regard to child self-regulatory skills. We obtained teacher ratings on the same measures that were used with parents (i.e., ratings of Hyperactivity, Attention Problems and Aggression) and we tested children on four neurocognitive measures assessing aspects of self-regulation. Unfortunately, both of these methods ended up with some significant limitations which hinder the ability to accurately interpret the data. With regard to teacher ratings, the children were all rated within the normal range (T-scores in the 50 s) at baseline and as such any improvements also fell within the normal range and do not appear to have clinical significance. Additionally, in those instances where teachers reported reductions in behaviour problems post-intervention and at follow-up, they also reported these in the waitlist-to-baseline period where no intervention occurred; and as such one cannot be certain that any improvements are related to intervention effects. It will be important in future studies for more clinically severe samples to be recruited including children where both parents and teachers report significant elevations in behavioural ratings. This will be challenging as parents and teachers often provide quite different reports on child behavior. The reasons for this could be environmentally driven, influenced by rater interpretation, or both^30,31^.

With regard to the neurocognitive testing, the children completed the same task at each time point as neurocognitive measures with alternate forms are not available in the preschool age group. This was somewhat controlled by all children in both groups doing the same measures each time and by controlling for age within the analyses. However, the only task that showed significant improvement post-intervention was Comprehension of instructions in the Triple P group, where again these children also showed significant improvements following the waitlist period and so the improvements cannot be attributed to the intervention with any certainty. The 12-month follow-up data are more challenging to interpret as both the ENGAGE and Triple P group showed improvements in Comprehension of Instructions and Heads-Toes-Knees-Shoulders. This could be attributed to practice effects or it may be that improvements in neurocognitive functions take longer to become apparent and that only by 12 months were treatment effects becoming apparent. Future studies will need to follow a non-treatment control group over 12 months to better ascertain whether these effects are simply practice effects. We did not do this in the current study as it was not deemed ethical for us to recruit at-risk children and not offer an intervention for 12 months. Additionally, a past study examining ENGAGE^8^ indicated that the behaviourally rated treatment gains over 12 months occurred above any beyond the natural reductions found in their comparison no treatment group, and as such this has already been ascertained with regard to treatment effects over time.

Additionally, the field is in need of neuropsychological tests for pre-schoolers that have alternative forms, as they do for adults, to enable better ability to retest participants over time within longitudinal studies.

## Conclusion

Despite the limitations discussed above, our results indicate that parents spending regular one-on-one time playing with their young children has ***the same*** positive effect on children’s behaviour as using behaviour management techniques which have a long history of being effective in managing child behaviour.

Thus, we now have an additional treatment option for young, at risk children that is enjoyable, low cost, easily accessible, and associated with long term maintenance of treatment gains. Although our current treatment options of medication and behaviour management are highly effective, they do not work for everyone and therefore having an additional, equally effective intervention available provides another treatment option for clinicians and families and may help those for whom the other interventions are not effective or palatable (particularly in the case of medication in preschool aged children).

## Data Availability

The datasets analysed during the current study are available from the corresponding author on reasonable request.
